# A Protein‐Based, Water‐Insoluble, and Bendable Polymer with Ionic Conductivity: A Roadmap for Flexible and Green Electronics

**DOI:** 10.1002/advs.201801241

**Published:** 2019-01-09

**Authors:** Firoz Babu Kadumudi, Mohammadjavad Jahanshahi, Mehdi Mehrali, Tiberiu‐Gabriel Zsurzsan, Nayere Taebnia, Masoud Hasany, Soumyaranjan Mohanty, Arnold Knott, Brent Godau, Mohsen Akbari, Alireza Dolatshahi‐Pirouz

**Affiliations:** ^1^ DTU Nanotech Centre for Intestinal Absorption and Transport of Biopharmaceuticals Technical University of Denmark 2800 Kgs. Lyngby Denmark; ^2^ Department of Electrical Engineering Technical University of Denmark 2800 Kgs. Lyngby Denmark; ^3^ DTU Nanotech Technical University of Denmark 2800 Kgs. Lyngby Denmark; ^4^ Laboratory for Innovations in Microengineering (LiME) Department of Mechanical Engineering University of Victoria 3800 Victoria BC Canada; ^5^ Centre for Biomedical Research University of Victoria 3800 Victoria BC Canada; ^6^ Centre for Advanced Materials and Related Technology University of Victoria 3800 Victoria BC Canada

**Keywords:** ecofriendly materials, fleco‐ionics, flexible displays, flexible electronics, human motion detection, nanomaterials, silk

## Abstract

Proteins present an ecofriendly alternative to many of the synthetic components currently used in electronics. They can therefore in combination with flexibility and electroactivity uncover a range of new opportunities in the field of flexible and green electronics. In this study, silk‐based ionic conductors are turned into stable thin films by embedding them with 2D nanoclay platelets. More specifically, this material is utilized to develop a flexible and ecofriendly motion‐sensitive touchscreen device. The display‐like sensor can readily transmit light, is easy to recycle and can monitor the motion of almost any part of the human body. It also displays a significantly lower sheet resistance during bending and stretching regimes than the values typically reported for conventional metallic‐based conductors, and remains fully operational after mechanical endurance testing. Moreover, it can operate at high frequencies in the kilohertz (kHz) range under both normal and bending modes. Notably, our new technology is available through a simple one‐step manufacturing technique and can therefore easily be extended to large‐scale fabrication of electronic devices.

## 1 Introduction

Protein‐based components that are electrically active can open new exciting avenues in electronics because of their flexibility, greenness, lightness, and abundance.^1^, ^2^ Indeed, the replacement of conventional metallic‐based electronic components with such materials could potentially transform otherwise hard and rigid electronics into flexible and wearable electronics.^1^ However, protein‐based electronics still require additional consideration to enable them to resist the many demanding scenarios in nature and within the human body, as most of them quickly disintegrate in liquids and in response to various chemical and thermal stimuli. Since ionic conductors (ionics) can generate many exciting electronic devices^3^, ^4^, ^5^, ^6^, ^7^ and many protein‐based materials can be transformed into such mediators of electricity^1^, ^8^, ^9^, ^10^, ^11^, ^12^ a possible avenue for remedying these bottlenecks is via manufacture of water‐insoluble, thermally, and chemically stable protein films with high ionic conductivity.

In simple terms, ionic conductors can be defined as materials that conduct electricity through the passage of ionic carriers such as Li^+^, Na^+^, K^+^, Cl^−^, Mg^2+^, and Ca^2+^. They have been utilized to yield flexible Li‐ion batteries,^13^ stretchable electroluminescent display devices^14^ and were recently used to pioneer the development of stretchable and transparent touchscreen devices, loud‐speakers, and actuators.^3^, ^4^, ^5^, ^6^, ^7^ Even though these newly published studies have pushed the field of electronics to a new high, they still present drawbacks that need to be addressed in the foreseeable future. Notably, most of these devices have been made from synthetic components, which over time can become hazardous for humans and the environment. Therefore, research and development (R&D) into flexible and ecofriendly (Fleco) ionics are still in their infancy. This is rather unfortunate because these conductors could potentially bridge the deep gulf that currently prevails between electronic devices and “Fleconess.”

Naturally derived biopolymers such as alginate, nanocellulose, starch, hyaluronic acid, and chitosan are possible alternatives to the manufacture of Fleco‐ionics;^15^, ^16^, ^17^ however, some of these materials have many mechanical shortcomings in a cross‐linked and solidified state.^18^, ^19^, ^20^ Other alternative materials include protein‐based materials such as keratin and collagen. They are low‐cost, flexible, and electrically active, but like most proteins, they also have low environmental stability and insufficient mechanical integrity. This limits their utility as substrates for touchscreens and electronic displays.^1^ Thus, material scientists and electrical engineers alike currently find themselves with few options in the quest for green ionics with long‐term operational stability.

Here, we moved into this uncharted territory by transforming the natural polymer silk into a Fleco‐ionic material with stable performance in both aqueous and chemically active environments. Indeed, silk have won the hearts of material scientists over the centuries due to it incredible mechanical properties.^21^ These attributes include a specific tensile strength that outcompetes conventional steel alloys and a mechanical toughness that is second only to spider silk among organic polymers.^21^, ^22^ In addition, to its remarkable mechanical properties silk fibroin can also conduct ionic currents due its protonic properties.^23^ Concurrently, a number of breakthrough studies have harnessed the properties of mineral‐based nanomaterials into multifunctional polymers to enable a wide range of applications within the fields of biomedical engineering, materials science, and mechanical engineering.^24^, ^25^, ^26^, ^27^

Advantageously, layered silicates are considered as green mineral‐based nanomaterials with low environmental impact and can improve the thermomechanical properties of various polymer based films.^28^, ^29^ In simple terms, laponite (Chemical formula: Na_0.7_Si_8_Mg_5.5_Li_0.3_O_20_(OH)_4_) is a unique layered silicate, manufactured from naturally occurring mineral sources, with a platelets thickness of about 1 nm and diameter of 25 nm.^25^ This mineral nanomaterial forms a colloidal suspension when dispersed in water, and in this state, it is surface renders a permanent negative charge by dissociating the sodium ion Na^+^ from the intercalated structures while the predominant MgOH provides positive charge on the rims. These dispersed structures increase the interaction with charged polymers and create a reinforcement effect by minimizing chain mobility.

**Figure 1:**
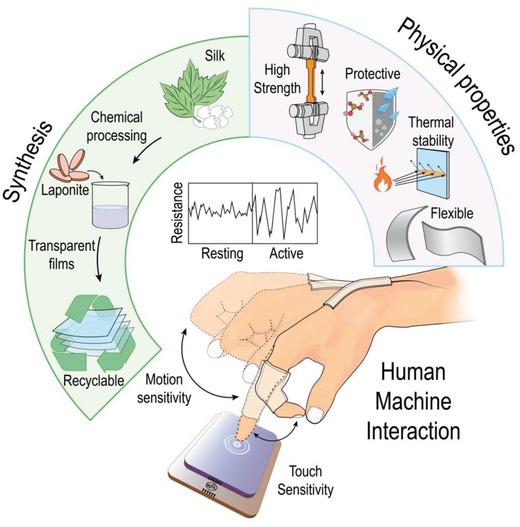
Illustration showing the preparation and properties of silk–laponite (SiPo) composite films.

Here, we tapped into both realms by embedding laponite into silk‐based thin films to generate an ionic conductor that exhibits a host of highly “sought‐after” properties in the field (**Figure** **1**). These properties include good crystallinity, transparency, mechanical strength, recyclability, optical transparency, electrical sensitivity, as well as chemical, thermal, and dimensional stability (Figure 1). Notably, the unmatched portfolio of concurrent attributes was achieved by only combining a few components through an ecofriendly and low‐cost procedure. By contrast, manufacturing their conventional counterparts is based on complex, time‐consuming, and expensive methodologies.^30^, ^31^ We demonstrated that our silk–laponite (SiPo) material is truly a Fleco‐ionic because it has a high electrical sensitivity after 2000 bending cycles and could be readily recycled through a simple and nonhazardous chemical process. We have also attempted to bring this exciting technology into real‐world applications by using it to prepare a sophisticated device that enables motion and touch sensitivity at the same time (Figure 1).

## 2 Results

### 2.1 The R&D of SiPo‐Based Fleco‐Ionics

**Figure 2:**
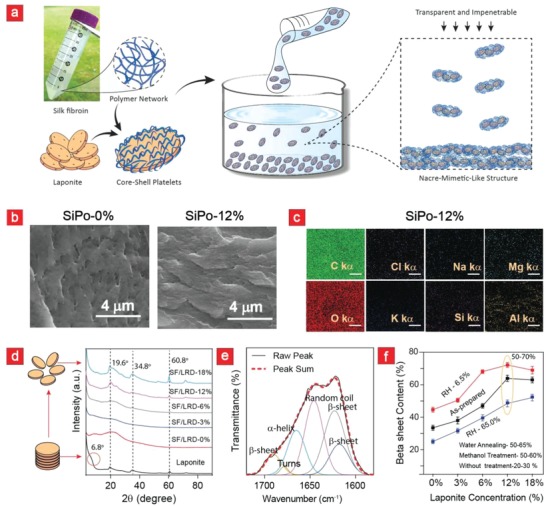
Synthesis and characterization of SiPO films. a) Schematic diagram showing the principles behind the manufacture of SiPo. b) Cross‐sectional SEM images and c) EDX characterization of SiPo films (Scale bar: 5 µm). d) XRD analysis, e) deconvoluted FTIR spectra of SiPo‐12% in the amide I region, and f) the FTIR‐associated β‐sheet content in the various SiPo films.

**Figure** **2**a presents a schematic of the SiPo film preparation. In brief, silk fibroin was mixed with laponite to yield a SiPo solution that was transformed into a freestanding film (100 ± 20 µm) through solvent cast drying. Silk fibroin is a polyampholyte with an isoelectric pH of approximately 4, and laponite displays a negative surface charge and a small positive rim charge.^24^ These two compounds can therefore potentially join into small agglomerates making it difficult to prepare a uniform and stable film.^32^ Indeed, in our preliminary experiments we observed a jellification of the SiPo film at acidic pH‐values, something most likely caused by a more positive charge distribution on silk at lower pH‐values, and the resultant more intensified electrostatic interactions with the negatively charged surface of laponite. We circumvented this by increasing the pH to generate a surplus of negative charge (decreasing zeta‐potential) on silk (Figure S1, Supporting Information). We also added potassium chloride (KCl) to the SiPo solution to further screen the electrostatic interactions. The combination of KCl and a basic pH ultimately enabled us to cast strong and uniform films for further downstream applications. The electrostatic interaction between laponite and silk fibroin was further confirmed through DLS measurements (Figure S1b, Supporting Information). From these results it is seen that the hydrodynamic diameter of silk fibroin (57.0 ± 8.0 nm) increased significantly with laponite incorporation. For instance, the mean diameter for SiPo‐12% was centered at 886.6 ± 57.8 nm, implying the formation of core–shell platelets due to electrostatic interaction as proposed in the scheme (Figure 2a).

To test the structural properties of the cast films, we performed a series of scanning electron microscopy (SEM) experiments. From these cross‐sectional images (Figure 2b; Figure S2a, Supporting Information) it is evident that the inclusion of laponite into the films could increase the layer‐by‐layer structure of the films albeit not to the same extent as reported in other studies.^25^ We attribute this to the formation of unwanted agglomerates despite optimization via pH and electrolytes. We also complemented the SEM imaging with energy‐dispersive X‐ray spectroscopy analysis (EDX) (Figure 2c; Figure S2b, Supporting Information) to demonstrate the presence of laponite within the SiPo films. From these EDX measurements important laponite trace elements such as silica (Si), sodium (Na), and magnesium (Mg) were clearly visible.^24^ The traces of aluminum (Al) were associated with background noise due to the aluminum stubs that were used for mounting the samples in the SEM chamber.

Next, X‐ray diffraction (XRD) studies tested the laponite dispersion within SiPo films (Figure 2d). Laponite RD powder had a broad diffraction peak at 2θ = 6.85° corresponding to the interplanar spacing between the basal plane of two parallel laponite sheets. The XRD studies show that laponite was almost completely dispersed within SiPo, i.e., this peak was not visible in the composites. In addition, the crystalline peaks of laponite at 2θ = 19.6°, 34.8°, and 60.8° are also clearly visible. On balance, this indicates that laponite was fully intact and well‐dispersed within the films. The stability of laponite was further validated via Fourier transform infrared spectroscopy (FTIR) characteristic laponite‐associated peaks at 1040 and 680 cm^−1^ were evident here (Figure S3a–c, Supporting Information).

In brief, silk fibroin is characterized by crystalline β‐sheets, α‐helices, and amorphous random coils. The β‐sheets are particularly important because they underlie silk's strength, rigidity, and stability.^21^ Therefore this structural characteristic of silk should be characterized to gain a deeper understanding of its structural integrity. Thus, we next used FTIR spectroscopy to determine the β‐sheet content in the various films (Figure 2e,f; Figure S3d, Supporting Information). We note a clear correlation between silk crystallinity and laponite incorporation. Notably, we could achieve a film crystallinity of almost 70% by simply increasing the laponite content to 12%. This is similar to what is achievable via conventional methanol and water wetting protocols, but without issues associated with an uneven macroscopic film morphology caused by film wetting and subsequent drying. In this regard, our surface roughness analysis showed a root‐mean‐square (RMS) value in the nanoscale range (Figure S4, Supporting Information). To further validate the results from Figure 2f, we also performed Raman spectroscopy on the SiPo films (Figure S5, Supporting Information). These results are similar to that observed from the FTIR spectra and confirm that laponite within SiPo films can facilitate the formation of β‐sheets.

In summary, our characterization studies established a new pathway for the manufacture of highly crystalline silk protein films via a simple and ecofriendly methodology. Next, we will further characterize the many incredible properties of these films and demonstrate their utility in a number of noteworthy applications in the forefront of the electronics field.

### 2.2 The Material Property Portfolio of SiPo

Flexible conductors based on serpentine‐configured metallic wires,^33^ carbon nanotubes,^34^ graphene,^35^ silver nanowires,^36^ gold nanosheets,^37^ conducting polymers,^38^ and metal oxides^39^ have sparked great interest in the field of electronics; however, many of these suffer from low transparency, environmental toxicity, the inability to operate at large frequencies, low biocompatibility, a high manufacturing cost, and apparent loss of electrical conductivity when undergoing several cycles due to mechanical fatigue.^37^, ^39^, ^40^, ^41^, ^42^, ^43^ In this section, we will demonstrate that SiPo simultaneously encompasses all of these properties without any unwanted tradeoffs.

**Figure 3:**
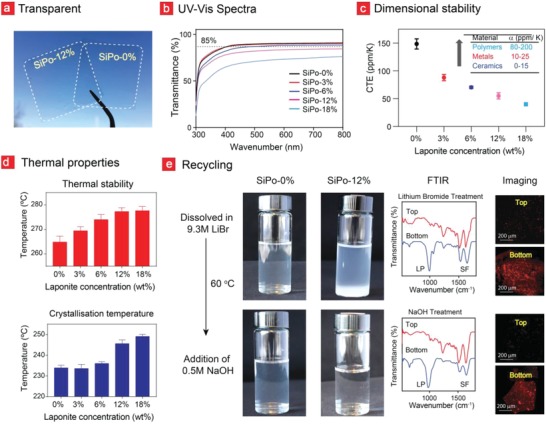
Transparency, thermal stability, and recyclability studies. a) Images showing the transparency of the films with and without laponite incorporation and b) UV–vis spectroscopy of the SiPo films. c) Dimensional stability of the films at elevated temperatures is displayed here in terms of the thermal expansion coefficient (CTE). d) The thermal stability and crystallization temperature of the films was examined with TGA and DSC. e) The chemical protocol behind the recycling of SiPo films are illustrated here together with FTIR analysis and fluorescence microscopic imaging of the SiPo supernatant and sediment after LiBr and NaOH treatment. The lower right photograph corresponds to the dialyzed silk sediment after NaOH treatment and centrifugation.

**Figure** **3**a,b shows that most of the SiPo films exhibit high transparency close to the required 85% transmittance for display devices (except for SiPo‐18%).^44^ Furthermore, the dimensional stability of the films are truly remarkable and outcompetes that of conventional polymers as evident from the coefficient of thermal expansion (CTE) results displayed in Figure 3c. The CTE coefficient decreased almost threefold as the laponite mass concentration increased from 0% to 18%. Our films also remained thermally stable up to 278 ± 1.6 °C and could reach a crystallization temperature of 249 ± 0.95 °C when incorporated with 18% laponite (Figure 3d; Figure S6a,b, Supporting Information).

The studies in Figure 3a–c were followed up by mass degradation experiments performed in various aqueous environments, which demonstrated an insignificant mass loss for SiPo films incorporating 12% and 18% laponite (Figure S7a, Supporting Information). We attribute the increased stability of our laponite incorporated silk films to the intensified laponite‐associated crystalline nature of the films, as the untreated silk films displayed low crystallinity and rapidly dissolved in water. Likewise, an increasing electrical conductivity from 1.7 × 10^−3^ to 4.6 × 10^−3^ S cm^−1^ was also observed as a function of film crystallinity (Figure S8a, Supporting Information). We also observed an improved water‐barrier property with increasing laponite content from water‐contact angle measurements, which is in accordance with the speculated layer‐by‐layer sedimentation of laponite within SiPo (Figure 2a). A decreasing water contact angle can be correlated to droplet water penetrating into the films—obviously if the contact angle remained the same or changed slightly, because of water evaporation, the films would display a barrier against water (Figure S7b, Supporting Information).

To acquire a better understanding of the electrical properties of SiPo, the electrochemical impedance spectroscopic (EIS) response was further analyzed by fitting an equivalent circuit (Figure S8c,d, Supporting Information). The EIS response is adequately fitted using the equivalent circuit with low χ^2^ values of 10^−2^ and the standard deviation obtained for the extracted electronics components (Figure S8e, Supporting Information) are comparatively low for the SiPo‐12% and SiPo‐18% films, indicating successful device fabrication.

Mechanical testing was also performed on the SiPo films (Figures S9 and S10, Supporting Information) to clarify any possible link between laponite incorporation and mechanical reinforcement. These experiments show a significant tensile strength and Young's modulus reinforcement in SiPo as the nanomaterial content increased from 0% to 12%. Noteworthy, we experienced some brittleness issues with the 18% laponite incorporated films, which could be linked to a more intensified formation of silk–laponite agglomerates. Therefore, while the highest ionic conductivity and dimensional/thermal stability was with 18% laponite, we used SiPo‐12% film for the remaining studies. For the sake of convenience we hereafter simply refer to SiPo‐12% as SiPo.

Finally, we tested the mechanical and electrical durability of SiPo by bending it for up to 2000 times (Figure S11, Supporting Information). For these measurements we hand‐picked the highest quality films, and could conclude that SiPo remained mechanically intact after these bending cycles and exhibited an unchanged electrical conductivity To add another dimension to the highly sustainable nature of SiPo, we developed a safe and easy‐to‐use protocol for its recycling. In this direction, the SiPo films were first dissolved in 9.3 m LiBr at 60 °C to yield a solution consisting of two layers (Figure 3e). From FTIR and fluorescence microscopy it was evident that laponite granulates were present in both of these layers, accordingly, this process was incapable of delivering a complete separation of silk and laponite (Figure 3e; Figure S12, Supporting Information). We therefore added 0.5 m NaOH to further separate laponite (bottom) from the silk fibroin fibers (top) (Figure 3e). The dissolved SiPo solutions were finally centrifuged and then dialyzed to facilitate an almost complete separation between the heavier laponite and more lightweight silk fibroin as seen in the FTIR spectra and fluorescence images. The remaining traces of silk in the laponite sediments (as observed in the FTIR spectra) are attributed to a possible irreversible adhesion between silk fibroin and the nanomaterials.

### 2.3 A Fleco‐Ionic Touchscreen Made from Protein

**Figure 4:**
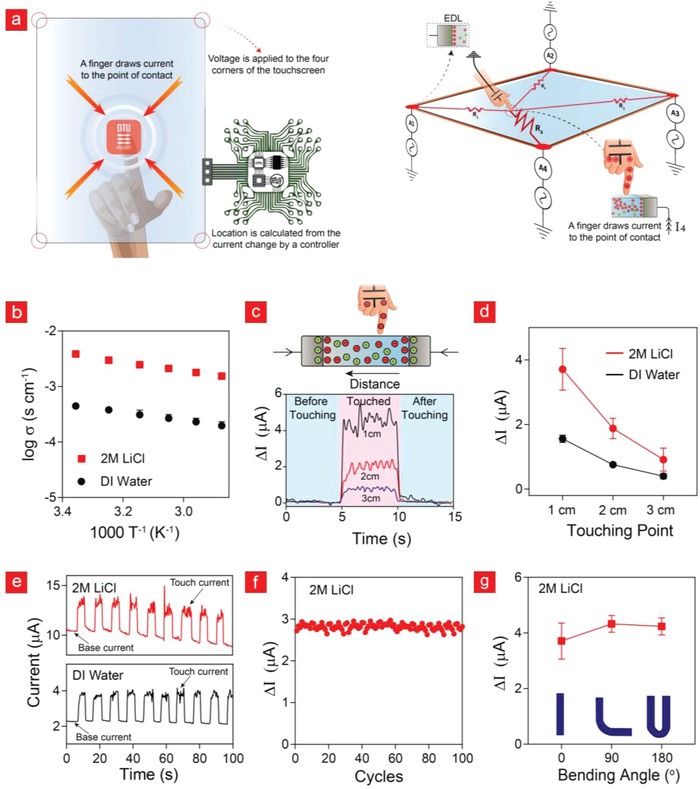
A touchscreen made from SiPo. a) Schematic representation of the working principle behind the surface capacitive touchscreens and their corresponding equivalent circuit. b) Ionic conductivity of SiPo‐12% film in different solutions. c) The current response before, during, and after screen touching. d) The electrical sensitivity at different touching points. e) The current response as function of time. f) The electrical sensitivity as function of touching cycle. g) The electrical sensitivity at different bending angles.

Touch‐panel screens are currently being used in various devices including portable computers, gaming consoles, mobile phones, human–machine–service interactions, and information kiosks.^45^ Recently, bendable and stretchable electronics have pushed this vibrant area to a new high, as flexible electronics presents an emerging technological goal in the R&D of wearable devices, flexible mobile phones, and foldable tablets. However, most of today's flexible touchscreens are based on synthetic polymers that have been coated with electronic‐waste generating, non‐biocompatible, and expensive indium tin oxide (ITO).^46^ Thus, our SiPo ionics meets all of the above‐listed shortcomings and could, therefore, become a next‐generation replacement for conventional ITO‐based screen‐technologies. To demonstrate this, we have used a capacitive touch‐sensing approach (**Figure** **4**a).^47^ Here, a uniform electrostatic field is generated across a conductive panel from which a human touch can draw a current and thereby induce a potential drop between panel and electrode. This potential difference then facilitates current flow from the electrode to the touch‐point and ultimately enables touch sensing via current detection.

In this section, we show that SiPo ionics can be transformed into high fidelity capacitive touch sensors by simply dipping them into a lithium chloride (LiCl) solution, as LiCl (≈10^−3^ S cm^−1^) can increase the ionic conductivity compared to DI water (≈10^−4^ S cm^−1^) (Figure 4b). More specifically, we used a 1D SiPo strip (dipped in LiCl) to manufacture a proof‐of‐concept Fleco‐based touchscreen. The 1D SiPo strip was then attached to two platinum (Pt) electrodes and an AC voltage of 0.5 V was applied across the electrodes using a function generator. Interestingly, a significant current increase was observed when this ionic panel was touched with a human finger. This response was monitored at 10 kHz and measured with an oscilloscope as shown in Figure S13 of the Supporting Information. The current difference (Δ*I*) from its base current is presented in Figure 4c. From the data presented in Figure 4c,d, it is evident that a human touch can generate a measurable current response with a magnitude depending on the touch‐point location. Therefore, one can use the magnitude of the current response to determine the coordinates of the touchpoint. Figure 4d shows that the slope is significantly steeper in LiCl than in DI water an indicator of a much better position‐sensitivity.

The touch current was very consistent in LiCl and DI water during cyclic touching (Figure 4e). Specifically, the higher ionic conductivity of our SiPo touchscreen in LiCl propagated a much higher base and touch current compared to DI water in these cyclic recordings. The touch response was also measured at high frequencies ranging from 10 to 40 kHz (Figure S13, Supporting Information), without any reduction in the operational capacity of the device, which makes our SiPo‐based electronics unique compared to many of their conventional metallic‐based counterparts.^43^, ^48^, ^49^, ^50^ To investigate the durability of the SiPo display, we next performed up to 100 cycles of touching and relaxing with a human finger, each cycle lasting 5 s, with the current response remaining unaffected (Figure 4f). Furthermore, the SiPo touchscreen was highly flexible, and the measured touch sensitivity remained almost the same even at a bending angle of 180° (Figure 4g), which is a “must‐consider” attribute in any wearable device. Thus, our SiPo touchscreen is well‐suited to become an interactive interface on wearable devices intended for use on flexing body parts.

### 2.4 Using SiPo for Human Motion Detection Sensing

Various motion capture imaging technologies are currently used to measure human motions. However, these approaches present a number of drawbacks related to their limited measuring range, low sensitivity, and impractical measuring scenarios. By contrast, flexible sensing systems that readily conform to the curvatures of the human body can measure motions from any part of the human body in any setting without any discomfort to the user. Such wearable sensors can gather biological information from athletes during games, soldiers in the battlefield, and musicians during concerts to improve their performances by helping them to gain the needed mastery of their subtle motions during strenuous activities.

**Figure 5:**
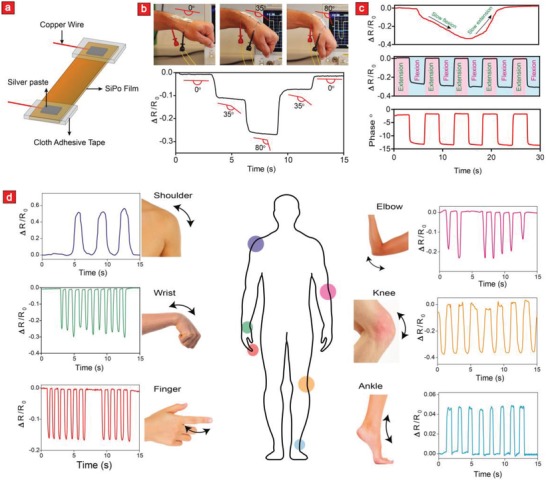
A SiPo‐based human motion detection sensor. a) Graphical representation showing the transformation of SiPo into a human motion detection sensor. b) Photographic images showing the bending motions of a human wrist equipped with a SiPo motion sensor and their corresponding relative electrical resistance responses during bending/unbending motions. c) Relative resistance changes during slow wrist movements (top), fast movement (middle), and the corresponding phase change during fast movements (bottom). d) Relative resistance changes for SiPo motion sensor attached to different parts of the body including the shoulder, wrist, finger, elbow, knee, and ankle.

Therefore, in addition to the touchscreen application mentioned in the previous section, we also tried to introduce SiPo as a new Fleco‐like material for human motion sensing. To this end, SiPo was immersed in LiCl and subsequently cut into 5 cm × 1 cm with both ends connected to copper wires using silver paste as shown in **Figure** **5**a. This Fleco‐ionic electrode was then attached directly to the moving parts of the body with an adhesive tape. The movements of the wrist were initially tested by bending it (from the extension to the flexion position) and monitoring the impedance changes through a precision impedance analyzer. The recorded impedance values were subsequently converted to resistance changes. In this respect, we observed a significant resistance decrease as the wrist was bent toward its flexion position (90°) (Figure 5b; Movie S1, Supporting Information). Specifically, the rate of resistance change depended on the motion type to a great extent. For instance, the wrist movement from 35° to 80° resulted in higher rates when compared with the same action from 0° to 35°. The observed resistance change also depended on the wrist speed, as a slow wrist movement results in a much smaller change compared to a faster motion (Figure 5c). For these reasons, our motion sensor can unravel both the dynamic nature of a wrist movement and the way that it is being moved—all within the same sensor. To demonstrate the cyclic measuring abilities of the proposed Fleco‐ionic sensor, we measured the square wave resistance during cyclic wrist movements with 2 s interval between each cycle (Figure 5c). These recordings confirm that the sensing performance of the sensor is not in any way compromised under cyclic body motions.

Next, we extended the motion‐sensing experiments to cover the locomotion of other important parts of the body such as finger, shoulder, elbow, knee, and ankle. Interestingly, the resistance behavior of our sensor deferred significantly depending on the human motion as is evident from the body part‐dependent signal patterns in Figure 5d. This trend is most likely caused by the previously speculated link between motion type/speed and electrical resistance (Figure 5b,c), and could, therefore, in down‐stream applications be employed to sense which part of the body is being moved. Such delicate sensing capacities can ultimately be harnessed into a wearable motion capture device that can unravel and quantify the complex motion of ballet dancers, musicians, surgeons, acrobats, and athletes in order to help them optimize their body movements when it really counts.

## 3 Discussion

In this work, we have showcased the many exciting properties of Fleco‐ionics, and utilized these exciting attributes to develop a wearable and green sensor. The Fleco‐like sensor was seamless in appearance, highly transparent and enabled human motion and touch detection capabilities. We accomplished this by transforming a protein‐based film made from silk (a natural ionic conductor) into a versatile and unusually stable material through the incorporation of inorganic, but yet green laponite. We coined this material SiPo and demonstrated that it was highly water‐insoluble as well as thermally and chemically stable this was attributed to a higher film crystallinity. Indeed, by simply increasing the laponite content in SiPo, we could significantly enhance its β‐sheet content and thus the crystallinity of the films.

To this end, recent studies have shown that 2D interfaces can enhance the folding of polypeptide chains in proteins into β‐sheets because of reduced conformation entropy.^51^, ^52^ This phenomenon implies that the conformational freedom of the protein is restricted by the interface, which ultimately limits the number of random protein configurations. Rather, it increases the likelihood of more ordered and crystalline configurations. Therefore, it is plausible to assume that the laponite facilitated silk crystallization is driven by a surface‐catalyzed fibroin folding into more ordered structures. This increased crystallinity in turn lowered the CTE coefficient, increased the thermal stability, and enhanced ionic conductivity without any undesirable transparency trade‐offs.

The linkage between thermal properties and crystallinity is a well‐established one,^44^ while the one between β‐sheet content and ionic conductivity is not as understood. Thus, we will focus on the latter in the following discussion. In this respect, a recent study by Pereira et al.^53^ established a similar interconnectivity between the degree of β‐sheets within silk films and lithium ionic conductivity. They hypothesized that the interaction between lithium ions and the dipole moments in the β‐sheet structure was a possible governing factor behind this observed relation. The β‐sheet dipoles were assigned an important role because they potentially could enhance the mobility of ions through a charge‐hopping mechanism. Generally speaking, higher temperature weakens the interionic bonds and enhances the free movement of ions, which increases the ionic conductivity of the ionic films. Silk proteins are naturally considered protonic conductors in presence of moisture. These trapped water molecules in the silk fibroin network interact with naturally‐occurring ion‐forming elements and hence generate ionic mobile charge carriers. Accordingly, the ionic conductivity of SiPo films is highly dependent on humidity and thus also the ambient temperature. In short, as the temperature increases the water content drops, and as results a decrease in ionic conductivity occurs.

The amount of accumulated electronic‐waste (e‐waste) is growing fast and doubled between 2009 and 2014 to reach a record high of 42 million tons per year.^54^ e‐waste from display‐based electronics such as personal computers, mobile phones, and portable electronics presents an especially huge environmental problem because the lifetime of such devices is decreasing rapidly as we speak.^55^ For these reasons, we sought to explore the possibilities of using transparent and ecofriendly conductors in electronics as an alternative to their problematic synthetic counterparts. To this end, we showed that SiPo is readily recyclable and can be recast again and again into new films. Along these thoughts, we demonstrated that SiPo is sustainable as well, as its electrical and mechanical properties were not compromised even after 2000 cycles. It could also maintain its touchscreen and sensing capacities following extensive cyclic usages. Overall, these studies add an extra ecofriendly dimension to an already spacious and impressive list of interrelated material properties.

We also demonstrated some unique operation capabilities of the developed Fleco‐like touchscreen: It could keep its touch sensing intact during various bending scenarios with an almost nonexistent performance hysteresis. Most synthetic displays do not possess such exciting properties. This issue is related to more compromised electrical connections in metal‐based conductors during high material strains. On the contrary, the conductance in ionic conductors relies solely on ionic electrolytes when the electrical medium is water. Therefore, the electricity can commence as long as the film is sufficiently hydrated, and the facilitator of charge locomotion, i.e., the polymeric backbone, remains operational.

The ability to operate at high frequencies and high sensitivity was at the very heart of our motion‐sensitive touch sensor. Indeed, it could operate in the kHz range, and it exhibited a motion and touch sensitivity of 40 and 180 ms, respectively. SiPo could, therefore, ultimately be used as a component in a number of devices in which high frequency operations and high sensitive are needed. Moreover, our SiPo‐based sensor can sense a wide‐range of human motions, as it takes about 300–500 ms for the human brain to process something in the real‐world into an consciousness experience and the duration of one of the fastest human motions (the eye‐blink) typically lasts 300 ms.^56^, ^57^

Another promising aspect of the technology developed herein is its low‐cost ($0.62/unit) and scalable manufacturing process. The low‐cost also makes it easier to extend this technology to cover large‐area interfaces on computers, television screens, commercial billboards, and various industrial machines. This will ultimately make the proposed sensors suitable for mass production without compromising the environment like their conventional counterparts.

## 4 Conclusion

Flexible and stretchable displays are currently being developed and marketed by Samsung and Sony, but these interfaces still do not provide combinatorial human–machine interactions. They also consist of highly expensive, toxic, and non‐recyclable components. Indeed, when such electronic devices are discarded they do not only possess a toxic threat for human habitats and those dwelling within them, but they are also increasing the demand for storage space that is increasingly difficult to find due to the growing global population.

In this study, we developed an alternative green option based on organic silk and inorganic green laponite for the display and wearables industry via Fleco‐ionics. The touchscreen displayed an accurate and fast response time even during various bending and stretching scenarios. We expanded this technology to also encompass human motion sensing of almost every part of the human body. Notably, we could distinguish the body part under motion and how they were moving. This sensing device was also highly durable and remained fully operational even after many locomotive cycles. Furthermore, the combinatorial mixing of touch and motion sensing could be used as a sophisticated and interactive tool for real‐time adjustments of motions during various performances or athletic training sessions.

## 5 Experimental Section

*Preparation of SiPo Thin Films*: Silk fibroin was extracted from Bombyx Mori silk cocoons (Wild Fibers, UK). Briefly, 10 g of sliced silk cocoons were boiled in an aqueous solution of 0.02 m sodium carbonate (Na_2_CO_3_, Sigma‐Aldrich, Germany) for 30 min, in order to remove all traces of sericin. The obtained silk fibroin fibers were dried at room temperature for 24 h. The fibroin fibers were then dissolved in an aqueous solution of 9.3 m lithium bromide (LiBr, Honeywell, Germany) at 60 °C for 6 h, and subsequently dialyzed against deionized (DI) water for 3 days. To remove any impurities, the resultant fibroin solution was centrifuged for 20 min (three times) at 12 000 rpm with the temperature kept at 4 °C.

In order to prepare SiPo films, the silk fibroin solution was diluted to 2.7% wt/vol in DI water. The pH was adjusted to 11 using 0.35 m ammonium hydroxide solution (NH_4_OH, 28.0–30.0%, Sigma‐Aldrich, Spain), followed by adding 10 × 10^−3^ m potassium chloride (KCl, Merck, Germany). Similarly, different concentrations (0%, 3%, 6%, 12%, and 18%) of laponite RD (LAP, BYK, UK) in DI water were prepared at pH 11, and incorporated into the silk solution. Finally, the mixture was casted onto a plastic petri dish and dried at 40 °C for 24 h.

*SEM and EDX Analysis*: The cross‐sectional images of the SiPo films were obtained by using an FEI Quanta 200 ESEM FEG scanning electron microscope (USA), equipped with a field emission gun electron source. In these experiments, the acceleration voltage and emission current was set at 10 kV and 10 mA, respectively. All SiPo films were cut, mounted on an SEM stub, and sputter coated with gold (10 nm) prior to the imaging process. Energy dispersive X‐ray spectroscopy was performed for elemental characterization of the respective samples before the gold sputtering. To this end, an Oxford Instruments X‐Max silicon drift detector with an 80 mm^2^ detection area (UK), which was connected to the SEM instrument, was used.

*XRD Analysis*: XRD characterization was performed with a HUBER G670 X‐ray powder diffractometer (Germany). The sealed X‐ray generator tube was operated at 40 kV and 40 mA. To this end, an imaging plate detection method in the Guinier geometry with a secondary monochrome and Cu X‐ray tube was utilized. XRD patterns were collected at room temperature with a 2θ ranging from 3° to 80° and a scan step size of 0.005° by using Cu Kα1 radiation (λ = 1.54056 Å).

*FTIR and Raman Spectroscopic Analysis*: FTIR spectra were acquired using a PerkinElmer Spectrum 100 FTIR spectrometer (USA), equipped with a diamond crystal attenuated total reflectance. The transmittance spectra were collected at 25 °C for SiPo films over the range of 4000–5000 cm^−1^ with 16 scans at a resolution of 4 cm^−1^. To examine the secondary structure of the silk proteins, the absorbance spectra of the Amide I region (1580–1750 cm^−1^) was deconvoluted using Origin Pro 2016 software (USA). To this end, the various absorption spectra were baseline‐corrected and fitted with Gaussian‐like peaks with a half‐bandwidth of 25 cm^−1^, using the PeakFit routine function of the Origin software. The FTIR measurements were also complemented with Raman spectroscopic analysis. The spectra were recorded from 100 to 3500 cm^−1^ at 25 °C for as‐prepared samples, using Thermo Scientific DXR Spectrophotometer (USA) with 455 nm excitation, 50× objective and a laser power of 5 mW. The crystallinity was then calculated using Origin Pro 2016 software from the deconvoluted spectra in the amide I region (1600–1800 cm^−1^).

*Zeta Potential and DLS*: The Zeta potential and DLS were measured with a Malvern Zetasizer ZS apparatus (UK), equipped with a 4 mW HeNe laser, operating at 632.8 nm. For Zeta potential, all measurements were performed at 25 °C for dilute solutions of silk fibroin and laponite and the pH was adjusted using sodium hydroxide (NaOH, Sigma‐Aldrich, Sweden) and hydrochloric acid (HCl, Emsure by Merck, Germany). The values are reported as the average of ten independent measurements. DLS analysis was performed three times for each sample. Laponite (0.5% wt/vol), silk fibroin (1.36% wt/vol), and the composites solutions were prepared in DI by mixing the required amount of materials in presence of 10 × 10^−3^ m KCl at pH11. All measurements (10 runs per measurement) were performed immediately after mixing at 25 °C without filtering.

*Surface Roughness*: The surface roughness was measured using a Bruker DektakXT Stylus Profilometer (USA) with a 2 µm stylus radius. A stylus force of 3 mg was applied and rastered over 1000 µm with measurements every 0.666 µm to generate a raw scan of the surface profile. Waviness was filtered out and RMS surface roughness was calculated using Bruker's proprietary Vision64 software (USA).

*Optical Transparency*: To determine the optical transparency of the SiPo films, UV–vis spectroscopic analysis was carried out using a Shimadzu UV‐2600 UV–vis spectrophotometer (Japan). Specifically, the spectra were collected by placing the SiPo samples in a film holder (P/N 204–58909) and using UVProbe software for interpreting the collected data. The transmittance spectra were then recorded using air as the reference in the interval between 200 and 800 nm. Finally, the transparency of the films was reported by measuring the percentage of transmitted light at 550 nm.

*Thermal Properties*: The thermal properties of SiPo films were characterized using a TA TGA Q500 Thermogravimetric analyzer (USA), a TA DSC Q200 differential scanning calorimeter (DSC, USA) and an RSA II Rheometrics solid analyzer (USA). Thermogravimetric analysis (TGA) was carried out inside a nitrogen‐saturated furnace, all the while the furnace temperature was increased linearly with a rate of 10 °C min^−1^ from 30 to 900 °C, and monitored the mass loss as function of temperature under a constant nitrogen flow at 60 mL min^−1^.

In addition, the differential scanning calorimetric (DSC) analysis was conducted by increasing the chamber temperature linearly from 25 to 280 °C with a heating rate of 10 °C min^−1^ under a dynamic nitrogen flow (50 mL min^−1^). To perform the DSC analysis, the SiPo films were encapsulated into Tzero aluminum pans (Switzerland), while an empty pan was used as a reference. The change in heat flow as a function of temperature was monitored, and the observed exothermic peak was interpreted as the crystallization temperature of the respective films.

A dynamic temperature ramp test was performed with Rheometric solid analyzer in order to determine the CTE. The test was conducted on 50 mm × 5 mm films, while the temperature was increased from 25 to 100 °C with a heating rate of 5 °C min^−1^ within a nitrogen atmosphere. Specifically, a constant force of 0.03 N was applied in tensile mode at a frequency of 1.0 Hz and constant strain of 1.0%. The linear expansion (Δ*L*/*L*) was collected as a function of temperature using Rhios software. CTE values were subsequently determined from the slope of the Δ*L*/*L* versus temperature curve in the range of 25 to 75 °C.

*Moisture Content Analysis*: The moisture content was determined by drying the SiPo films in an oven at 40 °C for 24 h. The weights of the samples before (*W*_o_) and after (*W*_d_) the oven drying process was measured using an electronic weighing balance with an accuracy of 0.001 g, and the moisture content (%) was subsequently calculated using the following equation

(1) $$\mathrm{Moisture}\;\mathrm{content}\left(\%\right)=\frac{W_{o}-W_{d}}{W_{o}}\times 100$$

*Swelling Study*: Swelling measurement was performed by immersing the SiPo films in DI water at room temperature. Initial weight (*W*_o_) and the weight at each time point (*W*_t_) were measured and the swelling ratio (%) was calculated using the following equation

(2) $$\mathrm{Swelling}\;\mathrm{ratio}\left(\%\right)=\frac{W_{t}-W_{o}}{W_{o}}\times 100$$

*Chemical Stability*: Chemical stability of the samples was assessed in DI water and different pH conditions (pH 2 and pH 11). SiPo films were immersed into the respective solution at room temperature and dried at each time point. The initial dry weight of the samples *W*_o_(d) as well as the dry weight at each time point *W*_t_(d) were recorded after 6, 12, 24, 72, 120, and 168 h, and the weight loss was calculated according to the following equation

(3) $$\mathrm{Weight}\;\left(\%\right)=100-\left(\frac{W_{o}\left(d\right)-W_{t}\left(d\right)}{W_{o}\left(d\right)}\times 100\right)$$

*Water Contact Angle Study*: The water barrier property of SiPo films was estimated through contact angle measurements using a Biolin Scientific Holding AB Attension Theta optical tensiometer (Sweden), equipped with a high‐speed camera that enables high‐resolution real‐time surface wetting experiments. To study the static contact angle change, 10 μL water droplets were gently deposited on the film surface, and photos were taken at different intervals for up to 600 s.

*Mechanical Tensile Testing*: The mechanical properties of the composites were measured with an Instron 5967 Universal Testing Systems (UK). Samples were cut into 30 mm × 2 mm rectangular shape and the thickness of each film were measured using a digital micrometer with an error 1 µm. The thickness varied in the range of 70–90 µm due to film inhomogeneity and therefore many samples (*n* = 5 to *n* = 10) of each combination were tested to reduce the standard deviation. The gauge length was set at 10 mm and the mechanical load was balanced prior to the test. The samples were pulled apart at the strain rate of 1 mm min^−1^ utilizing a 500 N load‐cell until the failure point was reached. Bluehill 3 testing software was employed to obtain a stress–strain curve, according to which, tensile strength, Young's modulus, and strain at break were determined. The Young's modulus was calculated in the strain range between 0.0% and 0.4% from the slope of the linear region of the stress–strain curve. To examine the influence of relative humidity on mechanical properties, the films were conditioned for 12 h in a desiccator at different humidity levels (6.5% ± 1.0% and 65.0% ± 1.0%), after which they were immediately subjected to mechanical testing. Relative humidity of 6.5% ± 1.0% and 65.0% ± 1.0% were generated using LiBr and sodium chloride (NaCl, Sigma‐Aldrich, Japan), respectively, and monitored using Omega RH85 handheld hygrometer.

*Recycling of SiPo Films*: To recycle the SiPo films, 500 mg of the samples was treated with 9.3 m LiBr at 60 °C for 2 h, followed by adding 0.5 m NaOH, which resulted in two separate phases. The mixture was then diluted 20 times with DI water and centrifuged at 12 000 rpm for 20 min. Notably, most of the laponite settled in the lower sediment, while silk fibroin remained in the solution. Both resulted solutions were then subjected to dialysis against DI water for 24 h, and dried at 60 °C. The presence of laponite and silk fibroin was confirmed through FTIR analysis. The supernatant and sediment were imaged by a fluorescence microscope to further examine how much laponite and silk were present in the respective phases. The fluorescence images and FTIR spectra were compared with pristine samples of laponite RD powder and freeze dried silk fibroin (Figure S12, Supporting Information).

*Ionic Conductivity Studies*: To study the ionic conductivity, SiPo films were immersed in an aqueous solution of 2 m lithium chloride (LiCl, Sigma, Germany) for 2 h. The EIS analysis was then utilized to determine the ionic conductivity of the films. EIS spectra were obtained from Gamry Potentiostat (USA) within a frequency ranging from 100 kHz to 10 Hz and applying AC voltage amplitude of 10 mV. All films were tested in semihydrated condition and the ionic conductivity was compared with the films prepared in deionized water. Samples of 1 cm^2^ were sandwiched between two stainless steel plates and clamped in order to fabricate a two‐electrode set up for the EIS measurements. The plates were then connected to the impedance analyzer and the set up was placed inside a jacketed reactor, equipped with water circulation system in order to control the temperature. This, two‐electrode set up was stabilized at each temperature for 5 min prior to the measurement, and the Nyquist data were collected using Gamry Instruments Framework software. The data were then curve fitted using EC‐Lab software from Bio‐Logic Science instruments to determine the solution resistance (*R*_s_). Finally, the ionic conductivity of the samples was calculated according to the following equation

(4) $$\sigma \;=\frac{1}{R_{s}}\;\times \frac{l}{a}$$

where σ is the ionic conductivity, *R*_s_ is the solution resistance, *l* is thickness of the sample, and *a* is the sandwiched area. The cyclic bending durability of the samples was also investigated for up to 2000 cycles by inspection, if the ionic conductivity remained the same as it was before the cycling process. At least four samples of each combination were used to make sure the observed phenomenon to this end was a general trend and not an experimental fluke.

The SiPo ionic touchscreen was fabricated by affixing two platinum plates on both ends of a 5 cm × 1 cm SiPo ionic strip using silver epoxy paste with the distance between the plates being kept at 3 cm. Prior to the touchscreen tests, the platinum plates were connected to a Kesight MSOS104A mixed signal Infiniium series Oscilloscope (USA) through a N2820A 3 MHz/Custom (1 µA) high sensitivity current probe (2‐channel) with a user defined resistor tip (1 Ω). To apply AC voltage at different frequencies, a Hewlett Packard 3312A function generator (USA) was connected to the 1D touchscreen. AC current was obtained from the oscilloscope by applying an AC voltage ranging from −0.5 to 0.5 V at different frequencies between 10 and 40 kHz, and the resulting current change from the finger‐touch was subsequently recorded. The absolute current and peak envelope of measured AC current was extracted using MATLAB R2016a, and the current difference (Δ*I*) was then calculated by subtracting the baseline current from the touch current. The current response to different touch‐point location and bending angles of the screen was essentially evaluated through the same procedure.

*Characterization of the Human Motion Sensor*: The motion sensing device was fabricated by connecting copper wires to both ends of a 1 cm wide SiPo ionic strip using conductive silver epoxy paste. Specifically, motion sensors of different length (from 3 to 6 cm) were attached to various moving parts of the body such as finger, wrist, shoulder, ankle, elbow, and knee, by means of cloth adhesive tape. Prior to participation, informed consent was obtained from the volunteer in all experiments. The working principle of the sensor relied on real‐time monitoring of resistance changes in response to body movements, which was monitored with an Agilent 4294A precision impedance analyzer (USA) operated at 10 kHz by applying an AC voltage ranging from −0.5 to 0.5 V. Relative resistance (Δ*R*/*R*_0_) was then calculated from the measured values, where Δ*R* is the difference between the resistance at the beginning (*R*_0_) and differing measuring scenarios.

## Conflict of Interest

The authors declare no conflict of interest.

## Supporting information

Supplementary

Supplementary
