# Supplementary material for: A Protein‐Based, Water‐Insoluble, and Bendable Polymer with Ionic Conductivity: A Roadmap for Flexible and Green Electronics
Source: Adv Sci (Weinh). 2019 Jan 9;6(5):1801241. doi: 10.1002/advs.201801241 (PMC6402400; doi:10.1002/advs.201801241)
Supplement: Supplementary file 1 — Supplementary [file ADVS-6-1801241-s002.pdf]

Copyright WILEY-VCH Verlag GmbH & Co. KGaA, 69469 Weinheim, Germany, 2019.

Supporting Information

for *Adv. Sci.,* DOI: 10.1002/advs.201801241

A Protein-Based, Water-Insoluble, and Bendable Polymer with Ionic Conductivity: A Roadmap for Flexible and Green Electronics

*Firoz Babu Kadumudi, Mohammadjavad Jahanshahi, Mehdi Mehrali, Tiberiu-Gabriel Zsurzsan, Nayere Taebnia, Masoud Hasany, Soumyaranjan Mohanty, Arnold Knott, Brent Godau, Mohsen Akbari, and Alireza Dolatshahi-Pirouz**

Supplementary Materials for

A protein-based, water-insoluble, and bendable polymer with ionic conductivity: A roadmap for flexible and green electronics

Firoz Babu Kadumudi[1], Mohammadjavad Jahanshahi[1], Mehdi Mehrali[1], Tiberiu-Gabriel Zsurzsan[2], Nayere Taebnia[1], Masoud Hasany[1], Soumyaranjan Mohanty[3], Arnold Knott[2], Brent Godau[4,5,6], Mohsen Akbari[4,5,6], Alireza Dolatshahi-Pirouz[1*]

[1]DTU Nanotech, Centre for Intestinal Absorption and Transport of Biopharmaceuticals, Technical University of Denmark, 2800 Kgs, Denmark.

[2]Department of Electrical Engineering, Technical University of Denmark, 2800 Kgs, Denmark.

[3]DTU Nanotech, Technical University of Denmark, 2800 Kgs, Denmark.

[4]Laboratory for Innovations in Microengineering (LiME), Department of Mechanical Engineering, University of Victoria, Victoria, BC, Canada

[5]Centre for Biomedical Research, University of Victoria, Victoria, BC, Canada

[6]Centre for Advanced Materials and Related Technology, University of Victoria, Victoria, BC, Canada

*Correspondence should be addressed to Prof. A. Dolatshahi-Pirouz - aldo@nanotech.dtu.dk

Zeta Potential and Dynamic light scattering (DLS) Measurements

The zeta potential has been measured through the phase analysis light scattering technique and the obtained values are plotted as a function of pH in the range between 3 and 11 (Fig. S1). Notably, laponite displayed a negative zeta potential across the entire pH range, whereas silk fibroin exhibited a positive zeta potential at pH 3 and negative values at higher pH values. Specifically, the zeta potential of laponite and silk fibroin reached -45.4 ± 1.5 mV and -14.2 ± 0.4 mV at pH 11, respectively, indicating excellent colloidal stability of both solutions. In addition, the presence of 10 mM KCl did not induce any surplus negatively charged moieties on laponite in an alkaline environment, while a slight increase was observed for silk fibroin with the zeta potential decreasing to -20.2 ± 0.5 mV. DLS measurement was performed to further confirm the electrostatic interaction between laponite and silk fibroin in presence of 10mM KCl at PH 11. The size distribution by intensity displayed an average hydrodynamic diameter of 30 ± 0.9 and 57 ± 8.0 nm for laponite and silk fibroin, respectively. The increased hydrodynamic size clearly shows electrostatic interaction between laponite and silk fibroin.

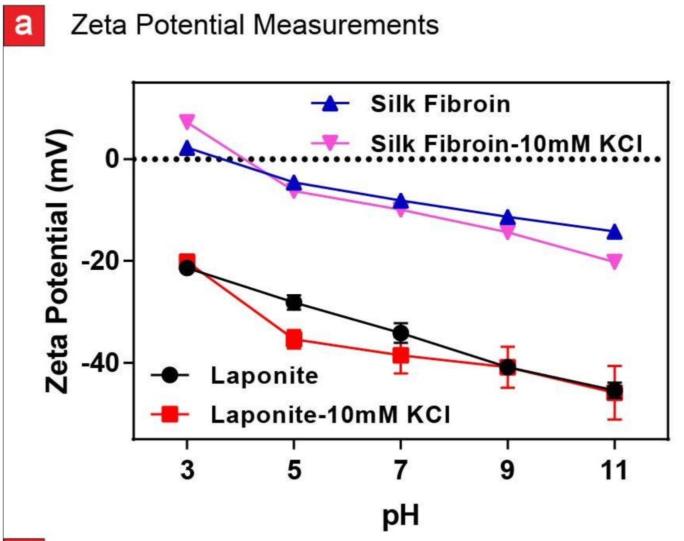

b DLS Measurements

S. No	Samples	Hydrodynamic diameter (nm)
1	Laponite RD	30.0 ± 0.9
2	Silk Fibroin	57.0 ± 8.0
3	SiPo-3%	335.1 ± 8.0
4	SiPo-6%	605.9 ± 25.8
5	SiPo-12%	886.6 ± 57.8
6	SiPo-18%	1266.3 ± 176.4

Figure S1. **Zeta Potential measurements**. a) Zeta potential of silk fibroin and laponite solutions with and without 10 mM KCl. b) DLS measurement for Laponite, Silk fibroin, and the composites.

Scanning electron Microscopy (SEM) analysis

In the case of control samples (silk casted at PH 7 without using KCL) and SiPo films with low laponite content, globular clusters were observed, while the incorporation of more laponite induced the formation of something reminiscent of layer-by-layer structures (Fig. S2a and Figure 2). In addition, EDX mapping of the control samples exhibited characteristic Kα X-ray emission of carbon and oxygen, whereas the SiPo-0% films exhibited potassium and chloride peaks as well. Minerals such as sodium, magnesium, silicon and aluminum, were also detected in the EDX spectra corresponding to laponite-incorporated samples. The mapping also reveals uniform distribution of the laponite elements, in accordance with a good polydispersity and minimal phase separation (Fig. S2a and Fig. 2c).

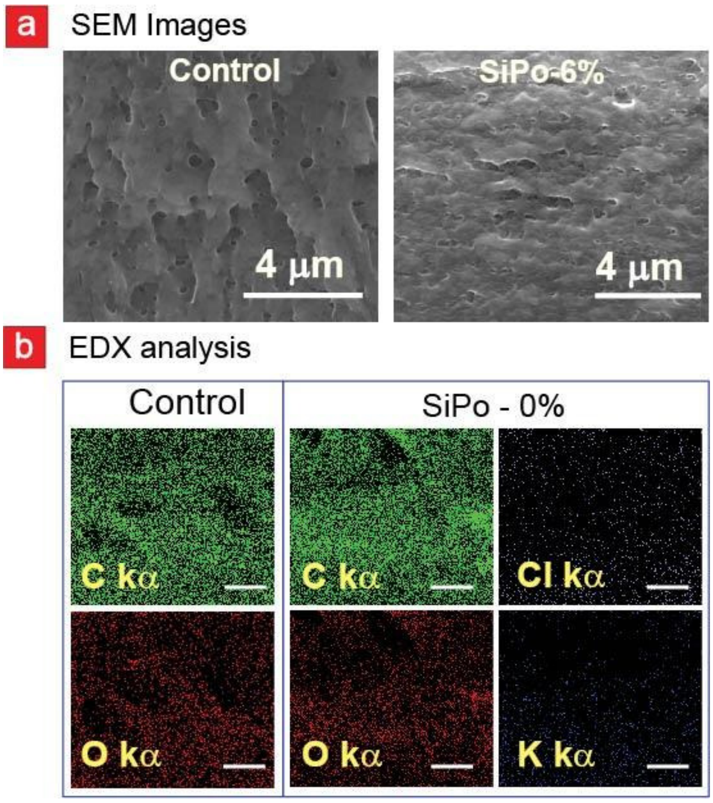

Figure S2. Morphological characterization. a) SEM analysis of control and SiPo-6% films b) EDX spectra of control and SiPo-0% samples (Scale bar: 5 μm).

Fourier transform infrared (FTIR) spectroscopy

FTIR spectra of SiPo films at different humidity conditions are shown in Fig. S3. Pristine laponite displays two major peaks at 1040 cm^{-1} and 680 cm^{-1} corresponding to Si-O-Si and Si-O stretching vibrations, respectively (Fig. S4). These peaks become more pronounced as the laponite concentration increased in accordance with both higher and well-dispersed laponite incorporation. What's more, the amide I band of the SiPo films was deconvoluted to determine the β-sheet content (Fig. S3d). The crystallinity (β-sheets content) of the films was then calculated by dividing the respective β-sheet content area by the total area using OriginPro 2016 (Fig. 2f). The β-sheet content increased under a humidity of 6.5% and reached a maximum of 70% for SiPo-12% films, yet it was found to decrease slightly under a humidity of 65%.

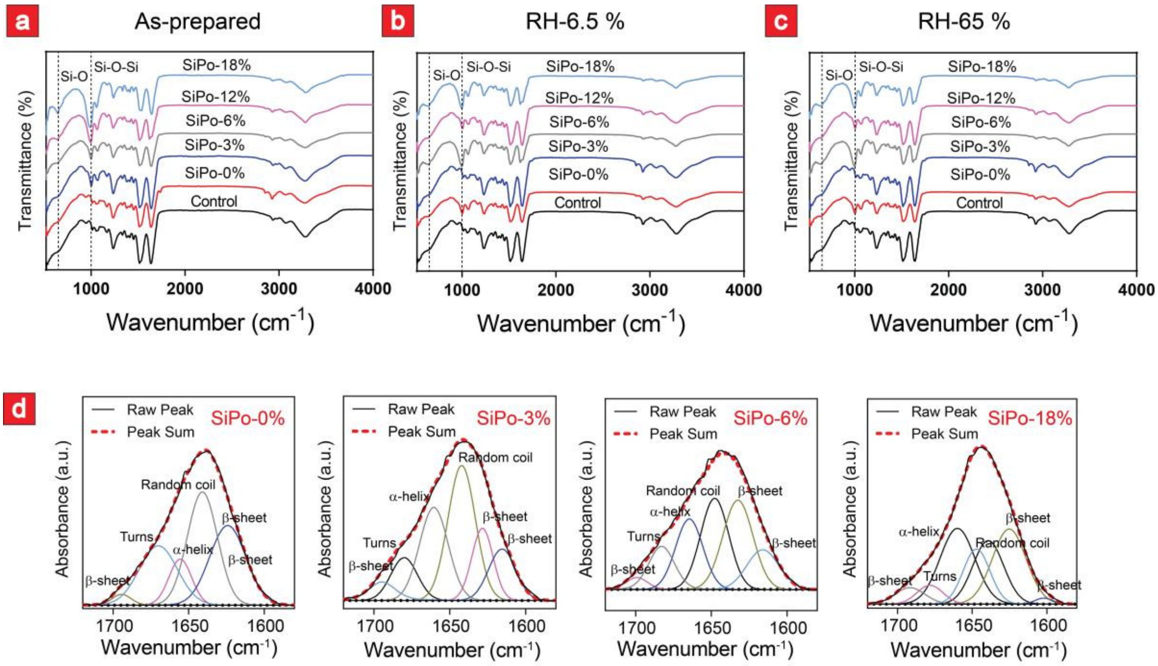

Figure S3. FTIR analysis. FTIR spectra of humidity conditioned SiPo films **(a)** as-prepared (**b**) 6.5±1% and **(c)** 65±1% relative humidity. (d) deconvoluted absorption spectra of as-prepared SiPo films in the amide I region.

Surface roughness

To examine the macroscopic uniformity in the Sipo films, the surface roughness was measured using profilometer. The average root mean square (RMS) surface roughness values obtained for the SiPo films are in the range between 10 and 20 nm (Fig.S4).

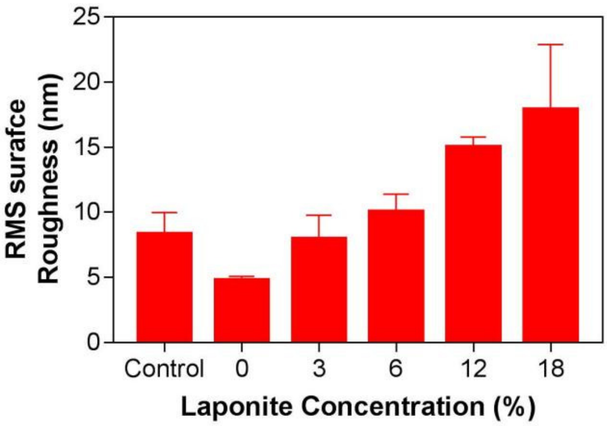

Figure S4. Surface roughness studies. Root mean square (RMS) surface roughness values of the various SiPo films

Raman spectroscopic analysis

To complement the FTIR results, the β-sheet content was also determined from Raman spectroscopy (Fig. S5). Absorption peaks at 1660 cm^{-1} and 1250 cm^{-1}, corresponding to amide I and amide III bands of silk fibroin, are evident here (Fig. S5). Notably, we detected a similar β-sheet content from these measurements as the ones retrieved from the FTIR spectra.

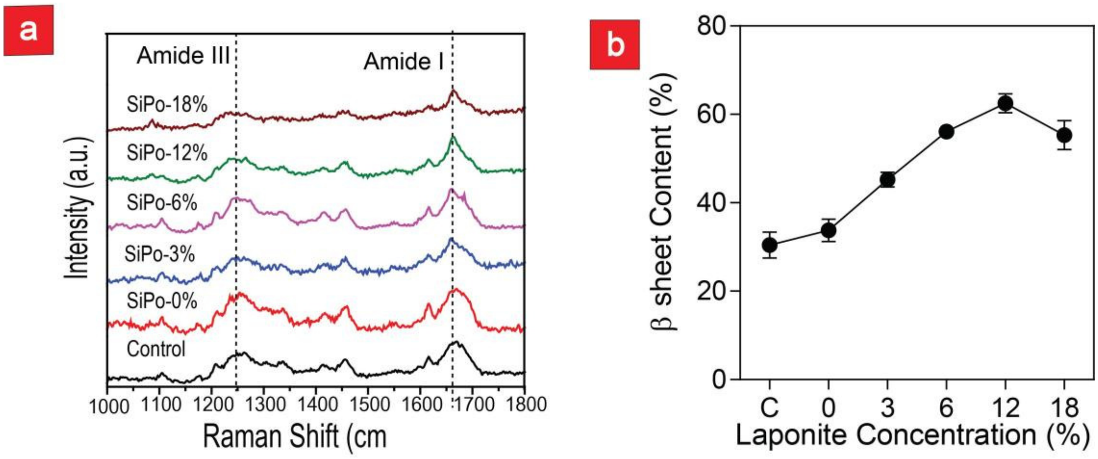

Figure S5. Beta content. Raman spectra of the respective samples are shown here together with their associated β-sheet content.

Thermal properties

Thermal stability of the composites was determined by thermogravimetric analysis (TGA) under nitrogen atmosphere (Fig. S6a). In this direction, an initial mass loss was observed for all samples below 200 °C due to the removal of bounded water. The large mass loss from 250 °C an onwards corresponds to the thermal decomposition of silk fibroin. Differential scanning calorimetric (DSC) analysis was also performed (Fig. S6b), and all samples showed endothermic behavior up to 150 °C due to water absorption, something that diminishes with increasing laponite concentration. SiPo-0% film possesses a glass transition temperature (T_g) of 178 °C, and displays a large endothermic peak at 227 °C as a result of non-isothermal crystallization of its amorphous chains. Interestingly, laponite incorporation resulted in an increased crystallization temperature, which corresponds well

with hypothesized capacity of laponite to guide the formation of β-sheets. Later, we investigated the swelling behavior of the SiPo films as function of varying laponite concentration (Fig. S6d). Since the casted control and SiPo-0% films were highly soluble in DI water, they were not included in these swelling studies. Maximum swelling ratio was reached after 20 min for all SiPo films, and the swelling ratio at saturation decreased from almost 100 % to 50 % as the laponite concentration reached 18 %.

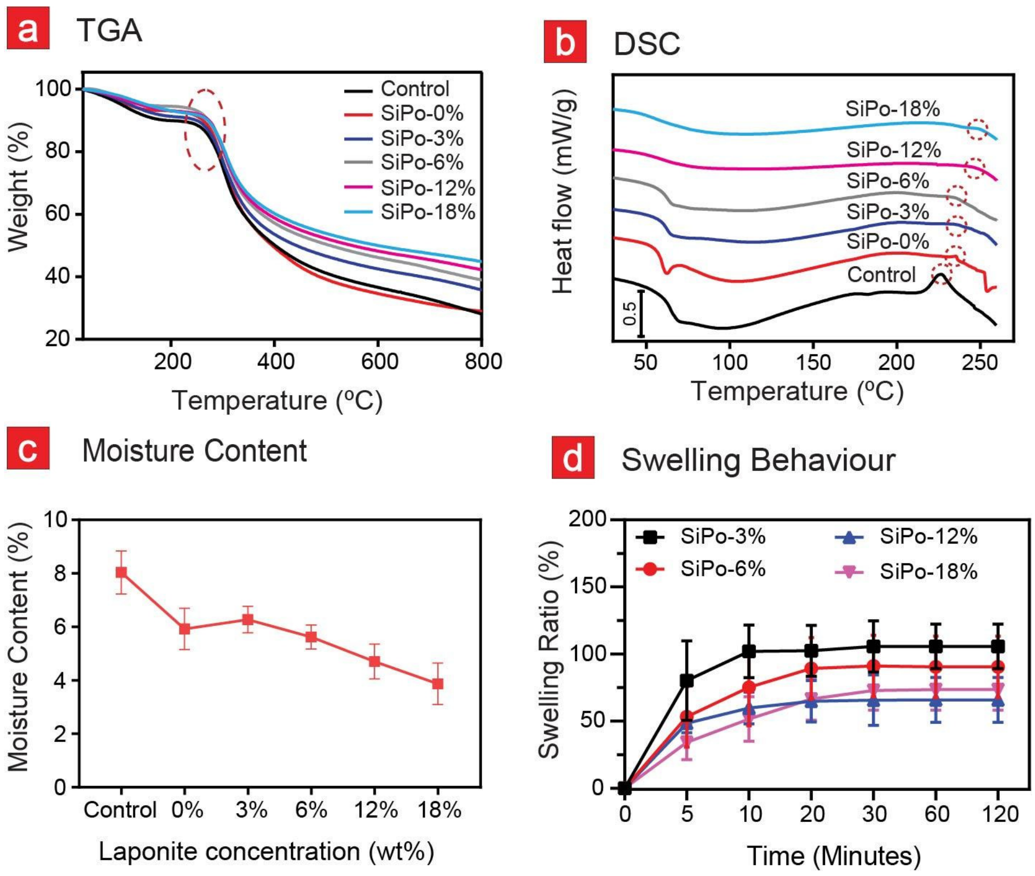

Figure S6. Thermal and water uptake properties. a) TGA, b) DSC, c) Moisture content and d) Swelling ratio of the various casted SiPo films.

Chemical stability assay

The major obstacle for the integration of natural biopolymers into electronic applications is their instability within chemically demanding environments. In the case of silk, its secondary structure,

which mainly consists of random coils and α-helices makes it highly soluble in aqueous solutions. Therefore, to enhance the stability of silk films at ambient conditions, researchers have over the years implemented promising methods such as methanol and water annealing treatments in order to increase the β-sheet content. In this study, we achieved this through laponite incorporation, as evident from our FTIR and Raman spectra's, as well as the increased stability of these SiPo films both within aqueous environments and under harsh chemical conditions (pH 2 and pH 11) (Fig. S7a).

Water barrier properties

The water barrier properties of the SiPo films were probed by measuring the water contact angle and its behavior over time (Fig. S7b). The idea here is that as the film absorbs water, the contact angle decreases. To this end, we found the initial contact angle of 55° for SiPo-0%, while its water repellency significantly increased with incorporation of laponite, reaching 130° at 12% laponite incorporation. Additionally, the contact angle of the SiPo-0% film significantly decreased over time and disappeared completely after 400 s. In contrast, the water droplet remained on the surface of the laponite-incorporated films and the respective water contact angle decreased by only 20° after 600 s for SiPo-12%. We speculate that this decrease can be attributed to the gradual penetration of droplet water into the films – with the penetration being more intense on the SiPo films with the least laponite incorporation. Altogether, our contact angle measurements on SiPo clearly demonstrated that its barrier properties increased concomitantly with laponite incorporation in accordance with other studies.

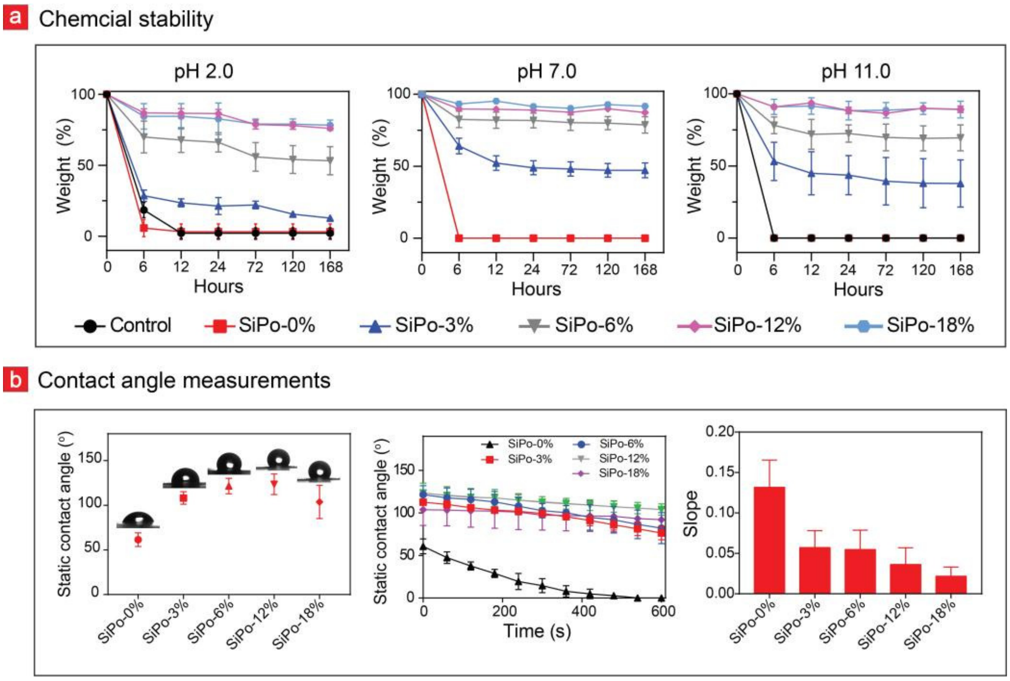

Figure S7. Chemical stability and wettability. a) The Chemical stability at pH 2.0, pH 7.0 and pH11.0 of the SiPo films are shown here. b) Static contact angle of the SiPo films was measured at different time points, and the slopes were retrieved from the resultant curves.

The intimate link between ionic conductivity and β-sheet content

The ionic conductivity of the semi-hydrated SiPo films, prepared in LiCl solution and deionized water were measured and correlated with the β-sheet content (Fig. S8). Semi-hydrated SiPo films are ionically conductive in deionized water and their conductivity lies in the range of 4×10^{-4} - 6×10^{-4} S cm^{-1}, something that increased almost 10-fold after LiCl treatment. These conductivity values correlate linearly to the β-sheet content within SiPo films. Electrochemical impedance spectroscopy (EIS) was employed to analyze electronic structure of SiPo films. From the Nyquist plots, all samples exhibit a depressed semi-circle in the high frequency region followed by a straight line in the low frequency range. This indicates well coordination of the equivalent circuit, shown in the Fig S8d, with the EIS response. This circuit is composed of a bulk resistance (R_b), a charge transfer resistance (R_1), a double layer capacitance (Q_1) and a bulk electrolyte capacitance (Q_2). Here, we introduced the constant phase element (Q) instead of ideal capacitance (C) due to the non-ideality of

the interface between the electrode and electrolyte in the practical EIS response. The corresponding values of each circuit element are provided in the Fig. S8e. Both resistance and double layer capacitance values are found to decrease with increasing laponite concentration, whereas the bulk resistance slightly increased with higher amount of laponite. The best fitted curves are obtained with the lowest χ^2 values of 10^{-2}. The large standard deviation in SiPo-3% and SiPo-6% samples are due to the inhomogeneous distribution of laponite in the films. On the other hand, the standard deviation for SiPo-12% and SiPo-18% are comparatively low and hence they are reliable for a device fabrication.

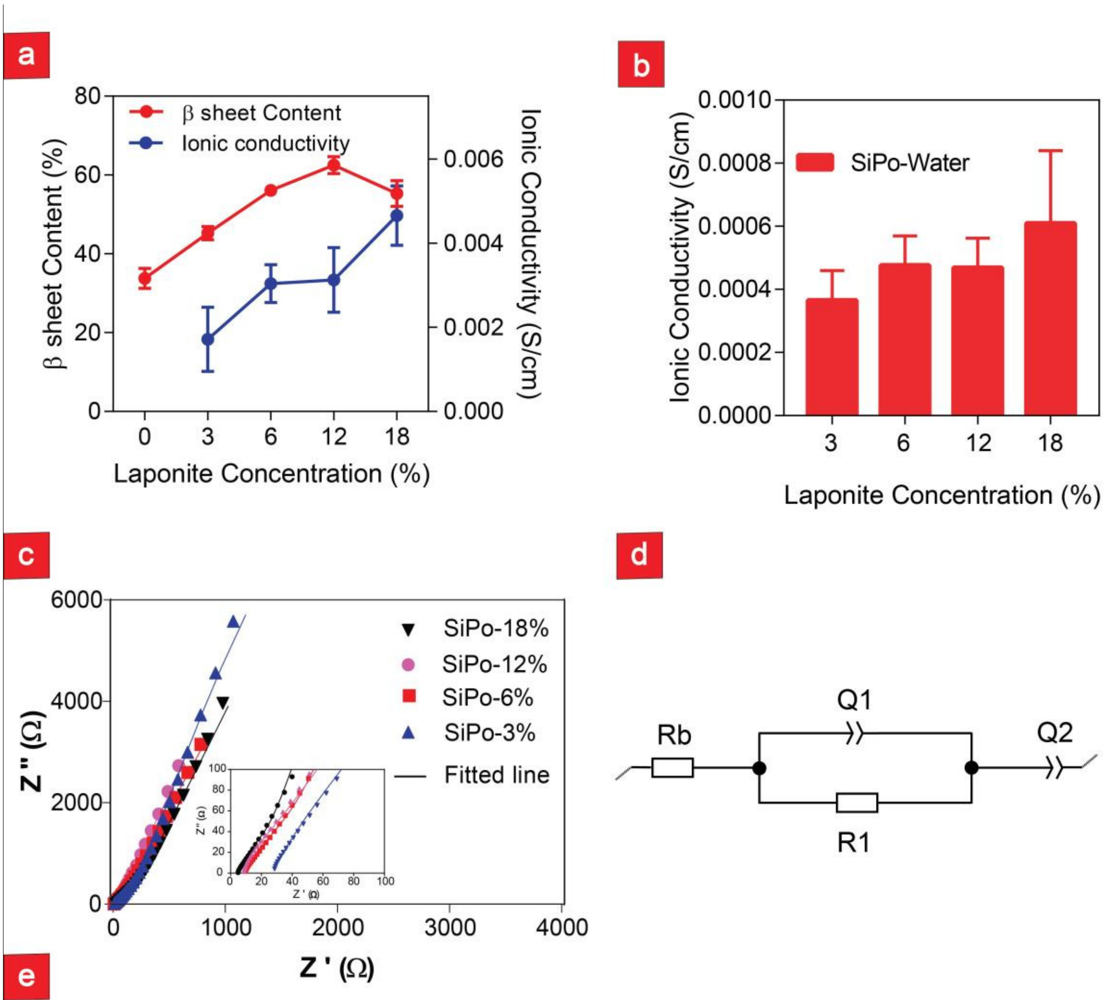

e

S.No	Samples	R_b (Ω)	R_1 (Ω)	Q_1 (μF)	a_1	Q_2 (μF)	a_2
1	SiPo-3%	13.4 ± 9.5	160.1 ± 222	14.3 ± 9.2	0.84	6.3 ± 5	0.86
2	SiPo-6%	10.7± 3.6	131.8 ± 24	9.6 ± 4.6	0.86	7.7 ± 6	0.86
3	SiPo-12%	7.0 ± 2.0	15.2 ± 2	4.6 ± 0.2	0.97	10.2 ± 3	0.83
4	SiPo-18%	5.5 ± 2.8	48.5 ± 7	3.7 ± 0.2	0.97	7.0 ± 2	0.87

Figure S8. **Crystallinity and ionic conductivity.** a) β-sheet content of the films and ionic conductivity of SiPo films in LiCl. b) Ionic conductivity of the samples in water. c) Electrochemical impedance spectra (EIS) of the SiPo films in LiCl (inset) enlarged portion of the EIS at high frequencies, d) equivalent circuit used for fitting the EIS response and e) the corresponding values of each circuit component.

Mechanical tensile properties

As-prepared SiPo films were examined in terms of tensile properties and compared with humidity-conditioned films (Fig. S9). As seen in the stress-strain curves, the SiPo-0% films undergo necking

after plastic deformation, whereas the control samples break during the plastic deformation regime. The necking region is present for the laponite-incorporated films as long as the concentration is below 12%, while it disappears by further addition of laponite. Importantly, the mechanical properties were highly sensitive to humidity, as SiPo samples at lower humidity levels (6.5 %) displayed more brittleness brittle compared to high humidity levels (65 %). As shown in Fig S8, by decreasing the humidity to 6.5%, the necking region of all the SiPo films completely disappeared, while the mechanical strength and young modulus increased with the strain at break decreasing from 1.9±0.5 to 1.4±0.3 for the SiPo-12% films. On the other hand, the behavior of SiPo films remains identical to what was observed for as-prepared samples at high humidity levels (65%), and these results clearly highlight the impact of moisture level on the mechanical properties of SiPo films. The mechanical properties were also measured for SiPo films, prepared at pH7 without adding KCl, and the corresponding data are provided in the Fig. S10. Here, the necking region was not observed at all, and the mechanical properties were comparatively lower than that of SiPo films prepared with KCl under alkaline condition. Moreover, the weaker interaction between laponite and silk fibroin in such neutral conditions restricted the laponite incorporation to 6%, as higher concentrations leads to more brittle films.

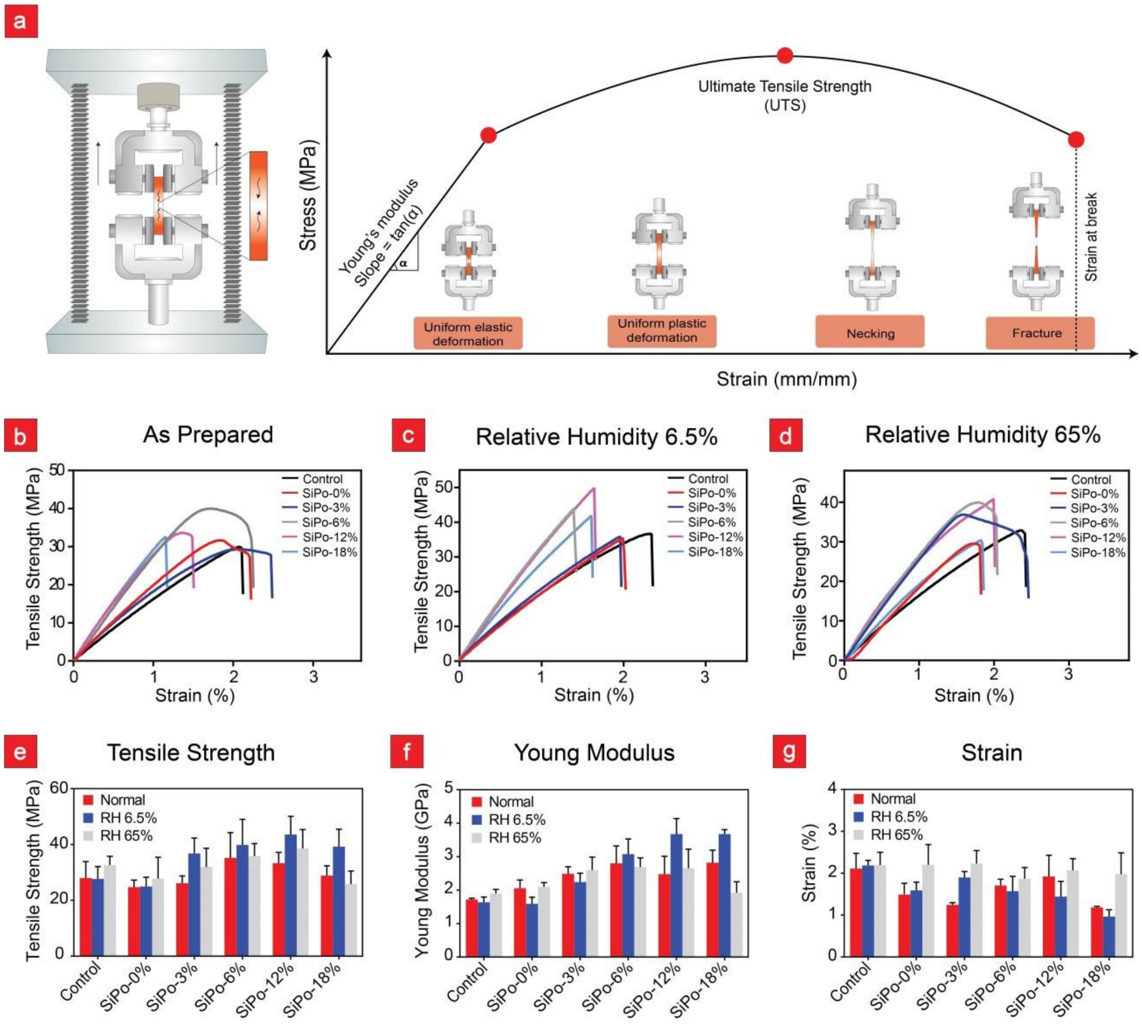

Figure S9. Mechanical properties. a) Schematic showing how the mechanical studies were performed. Stress-strain curves for the SiPo films b) as-prepared, c) conditioned at relative humidity of 6.5 % and d) conditioned at relative humidity of 65%. e) Tensile strength, f) Young modulus and g) maximum strain at break of the SiPo films.

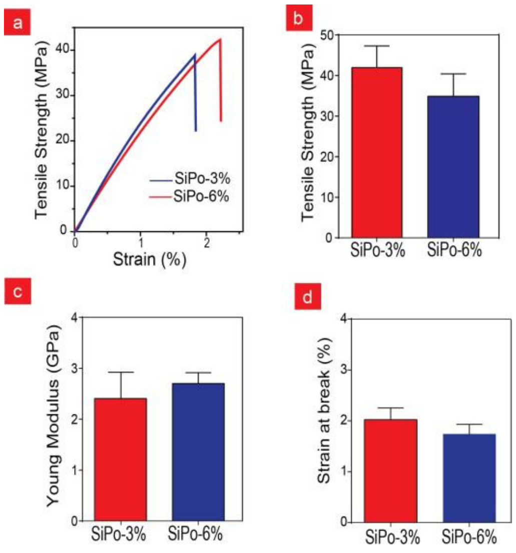

Figure S10. Mechanical analysis. a) Stress-strain curve, b) Tensile strength, c) Young modulus and d) maximum strain at break for SiPo films prepared at pH7.

The effect of cyclic bending on the conductivity of SiPo ionic films

SiPo-12% ionic films were bent to 180° periodically, to examine their bending durability in terms of their ionic conductivity. In order to prevent dehydration, films were re-immersed in LiCl solution prior to each measurement. Results showed that the ionic conductivity remained almost unaffected over 2000 cycles (Fig. S11).

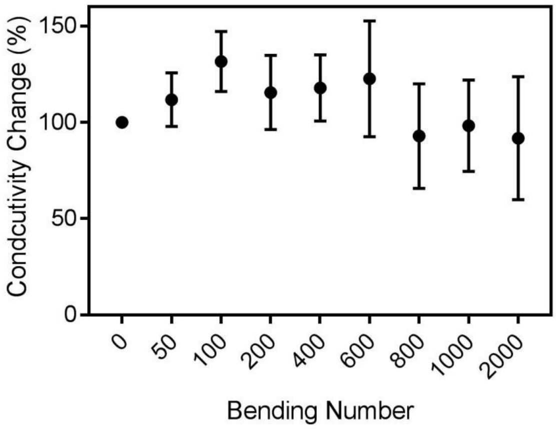

Figure S11. Electrical durability during bending cycles**.** Variation of ionic conductivity of SiPo films over 2000 bending cycles**.**

Characterization of pristine silk and laponite

Freeze-dried silk fibroin and pristine laponite powder were characterized using FTIR analysis in order to evaluate the recycled products.

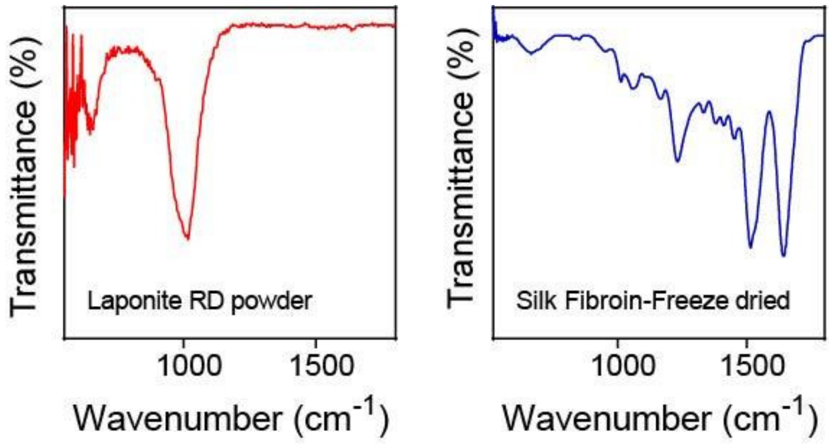

Figure S12. Recycling study. FTIR spectra of the pristine laponite powder and silk fibroin.

Current response of the touchscreen at different frequencies

The performance of the touchscreen was evaluated at different frequencies such as 10, 20 and 40 kHz. Here, an increased touch current difference (ΔI) was observed at higher frequencies, due to the decreased capacitive reactance (Fig. S13).

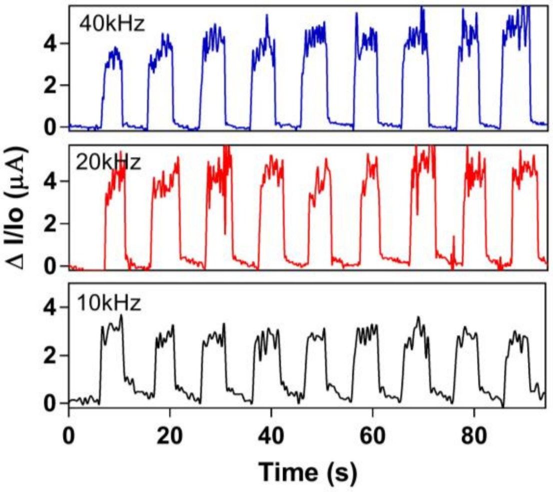

Figure S13. Touchscreen response. The Current response at different frequencies such as 10 kHz, 20kHz and 40kHz.
